# Interfacial Properties of the SnO/κ-Ga_2_O_3_ p-n Heterojunction: A Case of Subsurface Doping Density Reduction via Thermal Treatment in κ-Ga_2_O_3_

**DOI:** 10.1021/acsami.3c08841

**Published:** 2023-09-21

**Authors:** Payam Rajabi Kalvani, Antonella Parisini, Giovanna Sozzi, Carmine Borelli, Piero Mazzolini, Oliver Bierwagen, Salvatore Vantaggio, Kingsley Egbo, Matteo Bosi, Luca Seravalli, Roberto Fornari

**Affiliations:** † University of Parma, Department of Mathematical, Physical and Computer Sciences, Parco Area delle Scienze 7/A, 43124 Parma, Italy; ‡ University of Parma, Department of Engineering and Architecture, Parco Area delle Scienze 181/A, 43124 Parma, Italy; § IMEM-CNR, Institute of Materials for Electronics and Magnetism, Parco Area delle Scienze 37/A, 43124 Parma, Italy; ∥ Paul-Drude-Institut für Festkörperelektronik, Leibniz-Institut im Forschungsverbund Berlin e.V., Hausvogteiplatz 5-7, 10117 Berlin, Germany

**Keywords:** SnO/κ-Ga_2_O_3_ planar diode, capacitance−voltage (*C*−*V*) measurement, dual-frequency method, ultrawide band gap semiconductors, Synopsys Sentaurus-TCAD device modeling

## Abstract

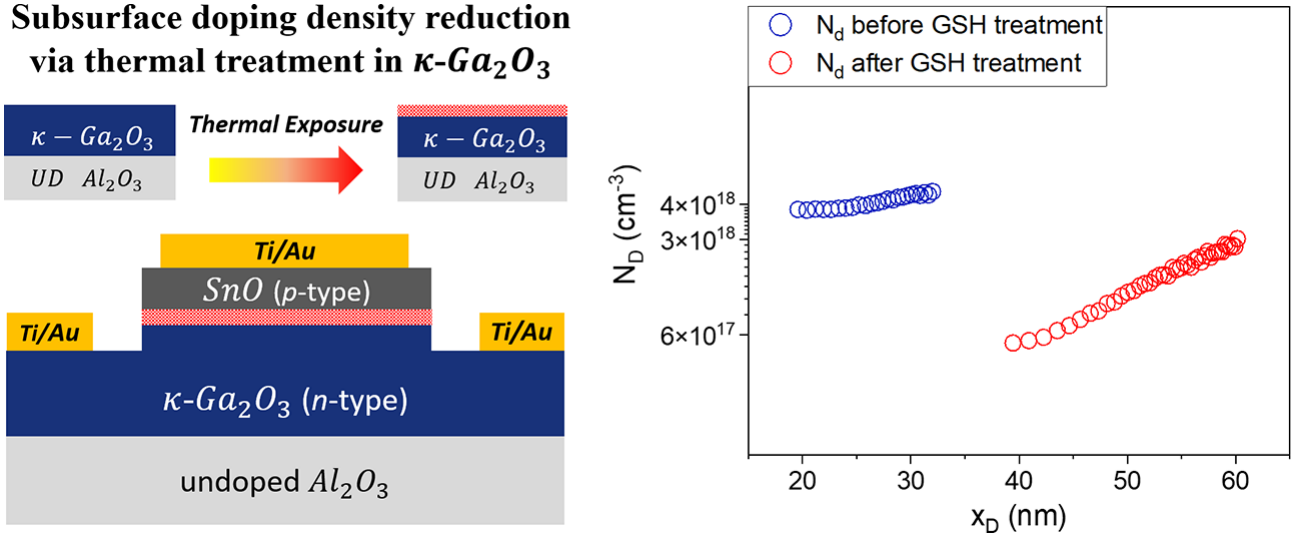

The interfacial properties of a planar SnO/κ-Ga_2_O_3_ p–n heterojunction have been investigated by capacitance–voltage (*C*–*V*) measurements following a methodological approach that allows consideration of significant combined series resistance and parallel leakage effects. Single-frequency measurements were carried out in both series- and parallel-model measurement configurations and then compared to the dual-frequency approach, which permits us to evaluate the depletion capacitance of diode independently of leakage conductance and series resistance. It was found that in the bias region, where the dissipation factor was low enough, they give the same results and provide reliable experimental *C*–*V* data. The doping profile extracted from the *C*–*V* data shows a nonuniformity at the junction interface that was attributed to a depletion of subsurface net donors at the n-side of the diode. This attribution was corroborated by doping profiles and carrier distributions in the n and p sides of the heterojunction obtained from the simulation of the measured *C*–*V* data by the Synopsys Sentaurus-TCAD suite. Hall effect measurements and Hg-probe *C*–*V* investigation on single κ-Ga_2_O_3_ layers, either as-grown or submitted to thermal treatments, support the hypothesis of the subsurface donor reduction during the SnO deposition. This study can shed light on the subsurface doping density variation in κ-Ga_2_O_3_ due to high-temperature treatment. The investigation of the SnO/κ-Ga_2_O_3_ heterointerface provides useful hints for the fabrication of diodes based on κ-Ga_2_O_3_. The methodological approach presented here is of general interest for reliable characterization of planar diodes.

## 1 Introduction

Nowadays, a major fraction of consumer and industrial electricity is processed by power electronic devices and instruments; therefore, the role of high-power electronics, and inherent losses, becomes increasingly important.^1−5^ Current power electronics face many technical challenges such as heat dissipation and overheating, limitations of the operation voltage, etc.^5−9^ There are also problems associated with the bad electrical contact that can lead to higher energy consumption or device breakdown due to the current crowding effect and localized overheating.^10^ It is important to note that about 13% of electricity is wasted in switching and conversion by conventional devices;^9^ therefore, development of new approaches and materials in high power electronics is an urgent and fundamental task. Ultrawide band gap semiconductors have emerged as good alternative for high power/low loss electronics. Specific physical characteristics of some of these materials, are high temperature operation, high switching rate, and very large breakdown field.^11−13^ Gallium oxide (Ga_2_O_3_) offers some advantages in comparison to the well-established SiC and GaN: wider band gap, between 4.5 and 5.3 eV (depending on the polymorph), large breakdown field near 8 MV·cm^–1^, and lower production cost.^14−16^ The specified value for the breakdown field was estimated through numerical calculations, while the highest value obtained experimentally for a dielectric heterojunction diode (metal/BaTiO_3_/β-Ga_2_O_3_) was 5.7 MV·cm^–1^,^17^ recently confirmed for a field effect transistor to be 5.5 MV·cm^–1^.^18^ It is reported that gallium oxide has five different polymorphs named α, β, γ, δ, and κ (or ε). So far, the monoclinic β-Ga_2_O_3_ has been the most investigated one, as it is thermodynamically stable. Therefore, bulk single crystals may be produced by melt growth techniques such as Czochralski (CZ), floating zone (FZ), vertical Bridgman, and edge-defined film-fed growth (EFG), which paves the way to production of high-quality substrates^19−21^ as well as to homoepitaxy. Many growth methods are available for epitaxial growth of Ga_2_O_3_ polymorphs such as metal–organic chemical vapor deposition (MOCVD),^22,23^ magnetron sputtering,^24,25^ mist-chemical vapor deposition (Mist-CVD),^26,27^ atomic layer deposition (ALD),^28,29^ pulsed laser deposition (PLD),^30^ halide vapor phase epitaxy,^31^ and molecular beam epitaxy (MBE).^32,33^ In spite of the numerous merits of the β-Ga_2_O_3_, there are concerns regarding its anisotropic thermal conductivity and easiness of cleavage.^8,14,34^ κ-Ga_2_O_3_ is the second most stable polymorph, and its orthorhombic cell has higher symmetry with respect to the monoclinic one, which may lead to easier epitaxial growth and novel heterostructures.^35,36^ Furthermore, it exhibits a spontaneous polarization along the [001] direction of the orthorhombic cell that could help to obtain high-density two-dimensional electron gases (2DEG) at the interface of κ-Ga_2_O_3_ based heterostructures,^37,38^ and thus a conducting channel for high-mobility field effect transistors,^12^ which is becoming one of the most intriguing topics of the literature on Ga_2_O_3_. Recently, κ-Ga_2_O_3_, was employed for fabrication of planar diodes^39^ and solar-blind UV–C photodetectors.^40^ It must be noted that this polymorph, which indeed has an orthorhombic crystallographic lattice, is often named ε because it shows a pseudohexagonal structure^41^ when investigated by X-ray diffraction. This derives from the presence of tiny orthorhombic domains, rotated 120° with respect to each other, produced by differently oriented rows of Ga octahedra and tetrahedra between oxygen planes in the heteroepitaxial layers. In the following, we shall use the name κ, more appropriate for identification of the microscopic structure of this phase. The domain walls, together with other vertically oriented planar defects, have been demonstrated to play a detrimental role for the in-plane electronic conduction of κ-Ga_2_O_3_ thin films resulting in a defect mediated conduction anisotropy (at least 1 order of magnitude lower resistivity in out-of-plane electrical measurements with respect to in-plane ones).^42^ Therefore, domain elimination (e.g., through epitaxially matched GaFeO_3_ substrates^21^) is an important issue for the further development of this polymorph.

A major drawback of all Ga_2_O_3_ phases comes from the lack of p-type conductivity, despite various doping attempts. Therefore, to fabricate diodes, one has to develop suitable Schottky contacts or p–n heterojunctions. So far the study of Schottky diodes and p–n junctions in planar or vertical configuration, based on the orthorhombic Ga_2_O_3_, is very limited, considering either inorganic^11,39^ or hybrid^43,44^ junctions. Nowadays, heterojunctions of metal oxides and other materials such as perovskites^45^ or chalcogenides^46^ are becoming popular subjects of investigation

In this work, tin monoxide (SnO) has been considered as a p-type material because of its high hole mobility,^47^ which is about 2 orders of magnitude higher than that of nickel(II) oxide (NiO)^48^ with the hole mobility of <0.1 cm^2^ V^–1^ s^–1^.^49^ The deposition condition of SnO has been optimized and vertical p-SnO/n-β-Ga_2_O_3_ diodes have been successfully investigated.^48^ As previously demonstrated for SnO deposited on β-Ga_2_O_3_, such a layer is stable after growth up to at least 300 °C under rapid thermal annealing (RTA) treatments performed in different background atmospheres.^47^ Consequently, the heterojunction of p-SnO/n-κ-Ga_2_O_3_ was investigated through the capacitance versus voltage (*C*–*V*) profiling, which is a popular method to characterize devices such as p–n junctions^48^ and Schottky diodes,^50^ metal–oxide semiconductors (MOSs),^51,52^ complementary MOSs (CMOSs),^53^ micro electro-mechanical system (MEMSs),^54^ magnetic-coded identification sensors (MISs),^55^ and metal-oxide-semiconductor field-effect transistors (MOSFETs).^56^

The *C*–*V* measurements provide information about the carrier type and density.^57^ In comparison to the Hall measurement, which provides average values of charge carrier density and mobility in a volume, the *C*–*V* analysis is suitable to explore the net dopant profile at the diode interface. On the other hand, the *C*–*V* investigation can be influenced by the temperature- and frequency-dependent contribution of deep levels. Further, the analysis of *C*–*V* characteristics is not straightforward and requires the identification of a correct equivalent circuit, which for the investigated structure includes the depletion capacitance *C*, the shunt conductance *G*, and a series resistance *R*_*e*_.

In this work, a methodological approach that permits us to reliably estimate the *C*–*V* data in the presence of nonuniform doping profile was tested and applied to the case of the SnO/κ-Ga_2_O_3_ planar p–n heterostructure. This approach is based on the comparison of single- and dual-frequency methods,^57−59^ the latter permitting a direct estimate of the capacitance *C* in the presence of both *R*_e_ and *G*. The *C* data were simulated by Sentaurus-TCAD device simulator to extract the impurity concentration versus depth in the planar diode, which provided new information on the technologically interesting SnO/κ-Ga_2_O_3_ heterojunction.

## 2 Experimental Section

To fabricate the planar SnO/κ-Ga_2_O_3_ diodes, a p-type SnO layer was grown by plasma-assisted molecular beam epitaxy (PAMBE) on a n-type Si-doped κ-Ga_2_O_3_ film deposited by metal–organic vapor phase epitaxy (MOVPE) on c-oriented sapphire. The thicknesses of κ-Ga_2_O_3_ and SnO layers were 550 and 150 nm, respectively. SnO resulted rather polycrystalline, with random in-plane orientation; then, lattice match does not seem to matter. Information about the XRD 2θ–ω scan of the SnO(001)/κ-Ga_2_O_3_ diode under study is reported in the Supporting Information of ref (39), where also more details about the fabrication procedures of the SnO/κ-Ga_2_O_3_ diode, its geometrical dimensions, and doping levels of the p- and n-type sides, independently investigated as single layers, are given.^39^ Further information about the result of room temperature Raman spectroscopy measurement of the SnO layer is given in the Supporting Information. The Hall hole density and mobility of the p-type SnO layer were 5.87 × 10^18^ cm^–3^ and 3.1 cm^2^/V·s, respectively.^39^ The Hall electron density, mobility, and resistivity of the n-type κ-Ga_2_O_3_ are given in Table 1. In this table, the electrical data of 4 n-type κ-Ga_2_O_3_ samples deposited under similar growth conditions are reported. Sample 1 is the one used to fabricate the diode under evaluation in this study (sections 3.1, 3.2, and 3.3), while the other three samples were used to verify the change of carrier density in κ-Ga_2_O_3_ (section 3.4) following thermal treatments. 250 nm-deep, square shaped SnO mesas with linear dimensions ranging between 75 and 200 μm were prepared by reactive ion etching. They were covered by square shaped, electron-beam evaporated 20 nm Ti/100 nm Au layers that serve as anode. The same type of metal stack was deposited around the mesa on the exposed κ-Ga_2_O_3_ layer to serve as cathode.This work is focused on 200 μm mesa structure.

**Table 1:** Electrical Properties of n-Type Si-Doped κ-Ga_2_O_3_ Films per Sample^a^

Sample number	Electrical resistivity (Ω·cm)	Hall mobility (cm^2^/V·s)	Hall density (cm^–3^)
Sample 1	0.416	4.06	3.69 × 10^18^
Sample 2	1.964	3.47	3.67 × 10^18^
Sample 3	1.636	1.14	3.35 × 10^18^
Sample 4	0.489	0.94	3.35 × 10^18^

a Error limits in the resistivity and Hall data are approximately equal to 5–10% and 10%, respectively.

Impedance spectroscopy and capacitance–voltage investigations were performed on the p–n junction by applying a small AC signal (50 mV) superimposed to DC bias voltage, in a range of frequencies where the dissipation factor is reduced. An impedance analyzer (LCR meter) HP 4284A operating in the frequency range 20 Hz-1 MHz was employed. The voltage range was adjusted between −3 and +0.3 V in steps of 0.01 V. We applied the AC signal at four different frequencies of 250, 300, 450, and 600 kHz, all lying in the range where the dissipation factor resulted lower than unity, and we recorded experimental capacitance values for all frequencies and voltages. This allowed us to investigate the influence of frequency on the *C*–*V* profile. We used the dual-frequency method to extract exact depletion layer capacitance *C*, series resistance *R*_e_ (or in series configuration: *R*_s_), and shunt conductance associated with the leakage *G* and compared them to the results of the parallel or series model at different frequencies to see where they are applicable. All values of the impedance, *R*_e_, *C*, *G*, and dissipation factor *D*, were experimentally determined as a function of applied voltage and frequencies. Finally, the doping density versus depletion depth was determined by three different approaches. In the first approach, an apparent profile was calculated directly from the proper numerical derivative of smoothed *C*–*V* data, in the second approach, it was determined by the Sentaurus-TCAD simulation technique, and in the third approach, the doping profile was determined from the Hg probe.

To reproduce the thermal conditions met by the κ-Ga_2_O_3_ during the SnO PAMBE growth, some samples were submitted to a growth simulation heating (GSH) cycle, which consists of the following steps: samples are placed in the same MBE chamber (vacuum = 10^–8^ mbar) and (i) heated up to 350 °C in a vacuum at a heating rate of 0.5 °C/s, (ii) exposed to an O-plasma treatment for 1 min (O_2_ flow = 0.18 sccm, plasma *P* = 200 W, *p* = 3 × 10^–6^ mbar), (iii) maintained at 350 °C for 22 min in a vacuum (*p* = 10^–7^ mbar), and (iv) cooled down in a vacuum to 100 °C and then to room temperature at a cooling rate of ≤0.5 °C/s. As for (ii), the exposure time of the layer surface to the plasma has been chosen considering the time needed in order to have full coverage of the κ-Ga_2_O_3_ layer by SnO, while step (iii) corresponds to SnO layer deposition time (during which the κ-Ga_2_O_3_ surface was not directly in contact with the plasma). Then, the samples were measured by a mercury probe and Hall effect to investigate the change of properties underneath the film surface. Hall effect and resistivity measurements were carried out, in van der Pauw configuration, on square samples with Au–Ti electrical contacts on the corners. A standard Hall effect experimental setup was used, applying a ±0.7 T magnetic field and DC current of ±50 μA. Typical uncertainty in the resistivity (5–10%) and Hall data (10%) can be considered on the basis of the reproducibility of the measure by varying the injected current and by taking into account the ratio between dimensions of the contacts with respect to the sample sides.

## 3 Results and Discussion

### 3.1 Equivalent Circuit and *C*–*V* Profile Assessments

**Figure 1:**
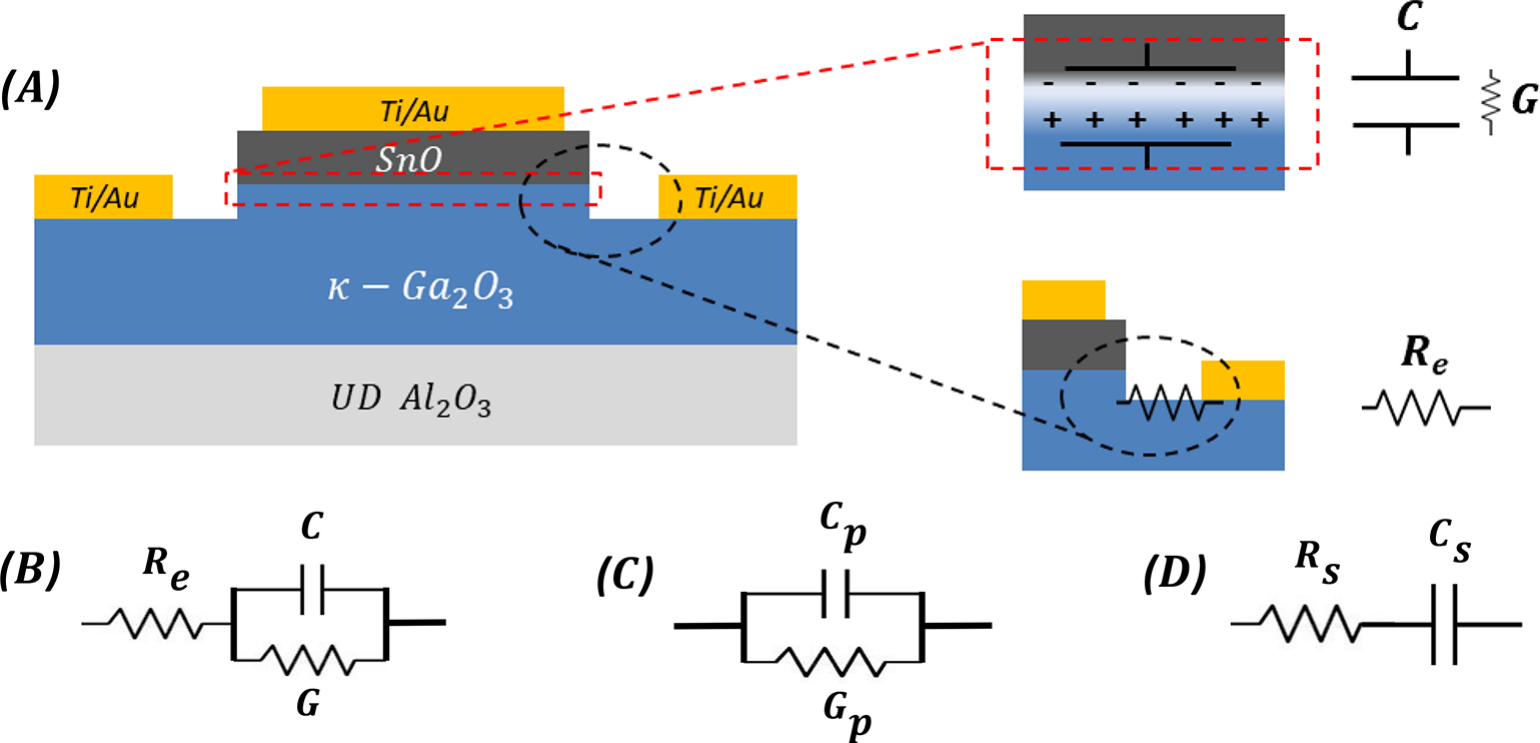
(A) Cross-sectional schematic diagram of planar diode with associated capacitance, series, and shunt resistances. (B) Equivalent circuit of the diode (three-element configuration): *R*_e_ represent the resistance in series with the junction; *C* and *G* represent the depletion layer capacitance of the diode and the shunt conductance associated with the leakage, respectively. (C) Parallel configuration. (D) Series configuration.

Defining the appropriate equivalent circuit for the diode is of great importance in order to correctly interpret the diode behavior through *C*–*V* profiling. Figure 1A, provides a cross-sectional schematic of the diode of this study, in which the series and shunt resistances as well as the capacity associated with the junction region are displayed. In Figure 1A,B, *R*_e_, *G*, and *C* represent the series resistance, conductance (which has an inverse proportional relationship with the dynamic resistance associated with the leakage), and capacitance in the three-element configuration, respectively.

Such a three-element circuit (Figure 1B), can be identified by AC impedance (admittance) measurements, which can be performed in two possible circuit configurations, series and parallel. The real and imaginary parts of the complex response of the device to an oscillating voltage are thus represented by either a shunt conductance and a capacitance, connected in parallel (*G*_p_, *C*_p_; Figure 1C), or resistance and capacitance in series (*R*_s_, *C*_s_; Figure 1D). However, in some studies the most appropriate equivalent circuit could require more than three elements, e.g., in ref (60), four-element equivalent circuit was considered with benefits in terms of more accurate simulation of *C*–*V* profile.

The adoption of the correct model associated with a given junction is extremely important, since an inappropriate choice can lead to the wrong determination of the carrier type.^57^ For instance, in the case of planar diodes, the series resistance given by the portion of material around the diode junction area has a high impact on the measurements and can drastically affect the estimate of the junction properties.^39^ The series resistance can influence the capacitance measurement,^60^ which itself can cause large errors in calculation of the doping profile,^61^ and built-in potential.^62^ Note that there are some physical and theoretical ways to reduce the impact of the series resistance on the *C*–*V* profile measurements.^58,59,62,63^

#### 3.1.1 Single-Frequency Approach: Parallel vs Series Model

**Figure 2:**
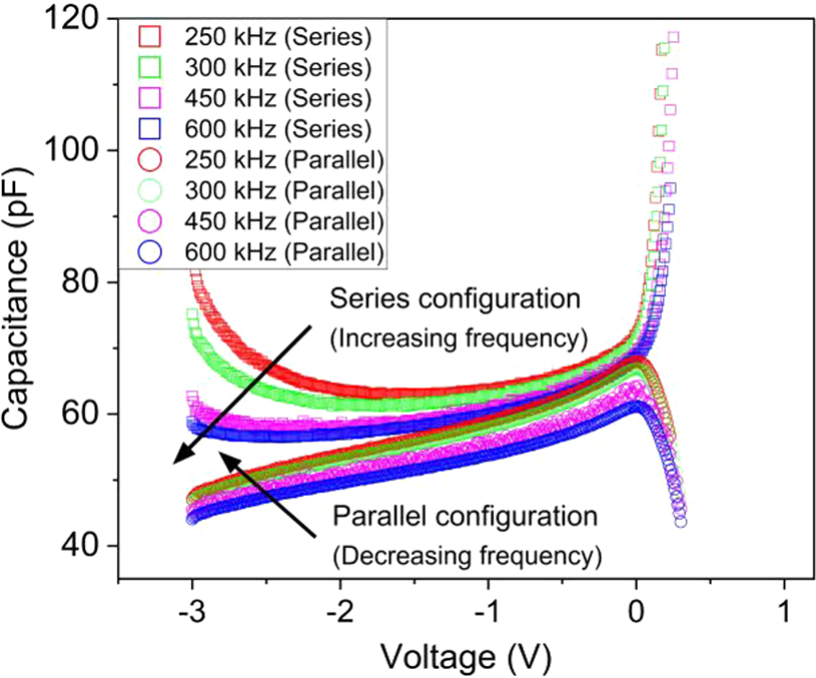
*C*–*V* profile at four different frequencies for both the series and parallel configurations. Squares are for series configuration; circles are for parallel configuration. Colors: red, 250 kHz; green, 300 kHz; pink, 450 kHz; blue, 600 kHz.

Series and parallel configurations at four different frequencies were applied to the three-element circuits of Figure 1 to identify the more reliable approach for the *C*–*V* profile extraction. It was observed that *C*–*V* curves taken at the highest frequency (600 kHz) for series configuration and at the lowest frequency (250 kHz) for parallel configuration were practically coincident, in a limited voltage range, as shown in Figure 2.

In order to study capacitance values in series and parallel configurations, eqs 1 and 2 were used. From eqs 1 and 2, it is understood that the measured capacitance, in series/parallel configurations, approaches the true depletion capacitance value (*C*), in the limit of high/low frequencies and negligible *R*_e_, respectively.
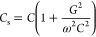
1
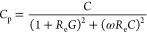
2In eqs 1 and 2, *C*_s_ and *C*_p_ are the capacitance values measured in series and parallel configurations, respectively, according to the elements defined in Figure 1B, *C*, *G*, and *R*_e_ are the true capacitance, conductance, and series resistance of the three-element model, respectively, and ω is the measurement AC frequency.

It is worth noting that *C*_s_ and *C*_p_ are linked according to eq 3:
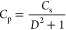
3where *D* is the dissipation factor defined by eq 4:
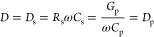
4where *D*_s_ and *D*_p_ are the dissipation factor in series and parallel configurations respectively, *R*_s_ is the series resistance in the series configuration (Figure 1D), *G*_p_ is the leakage conductance in parallel configuration (Figure 1C), and ω is the frequency of the applied AC signal.

It seems from Figure 2 that series and parallel configurations give the same capacitance, in a limited voltage range, at the highest and lowest frequency, respectively. In the Supporting Information, we also show that the corresponding nonlinear trend of 1/*C*^2^ vs reverse bias occurs in a limited voltage range. To confirm this conclusion, we also carried out a dual-frequency investigation.

#### 3.1.2 Dual-Frequency Approach

The dual-frequency method was originally proposed as a method to measure the true capacitance when the investigated device has a three-element equivalent circuit, Figure 1B,^58,59^ eliminating the contribution of the resistances from the measurement. Calculation of the capacitance through the dual-frequency method for the circuit of Figure 1B depends on the chosen series or parallel configuration for the measurement. In particular, the depletion capacitance (*C*) through the dual-frequency method is given by eq 5:
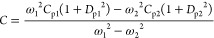
5where *C*_p1_ and *C*_p2_ are capacitance values and *D*_p1_ and *D*_p2_ are dissipation factors measured at the first and second frequencies (*ω*_1_ and *ω*_2_), for the parallel models, respectively. An equivalent equation for calculation of the true capacitance using a three-element circuit in a series configuration is given in the Supporting Information.

**Figure 3:**
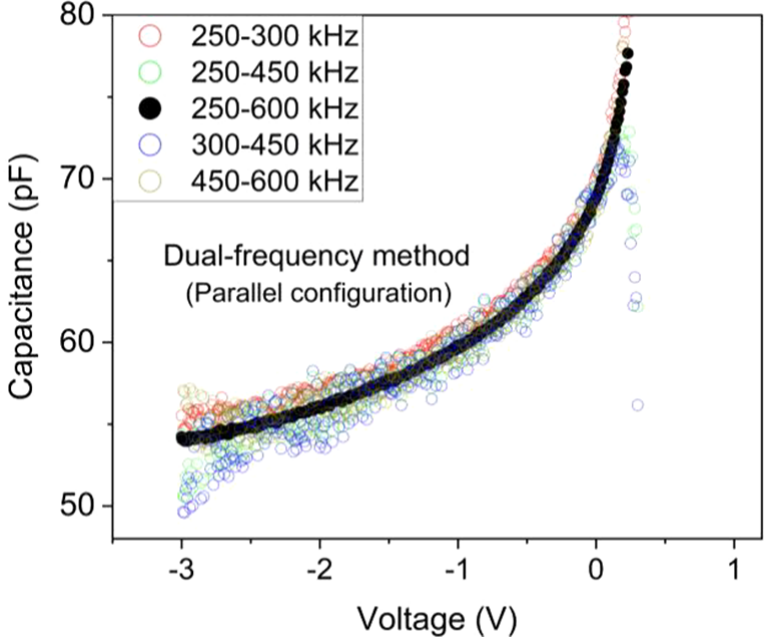
*C*–*V* profile of the dual-frequency method for the parallel model, Note the good matching of measurements at 250–600 kHz (full dots).

In this study, the dual-frequency method was applied for the following pairs of frequencies: 250–300 kHz, 250–450 kHz, 250–600 kHz, 300–450 kHz, 300–600 kHz, and 450–600 kHz. The associated *C*–*V* values for the parallel configuration are shown in Figure 3. For the frequency pair 250–600 kHz, the obtained *C*–*V* profile shows the best signal-to-noise ratio. In the Supporting Information, the same data obtained in the series configuration are also reported.

Thus, the pair of frequencies with the largest difference provides the most accurate values (lowest fluctuations), while by using pairs with smaller frequency gap, fluctuations in the *C*–*V* profile increase, which gives uncertain values for the capacitance.

#### 3.1.3 Correlations between the Single/Dual-Frequency Approaches and Dissipation Factor

**Figure 4:**
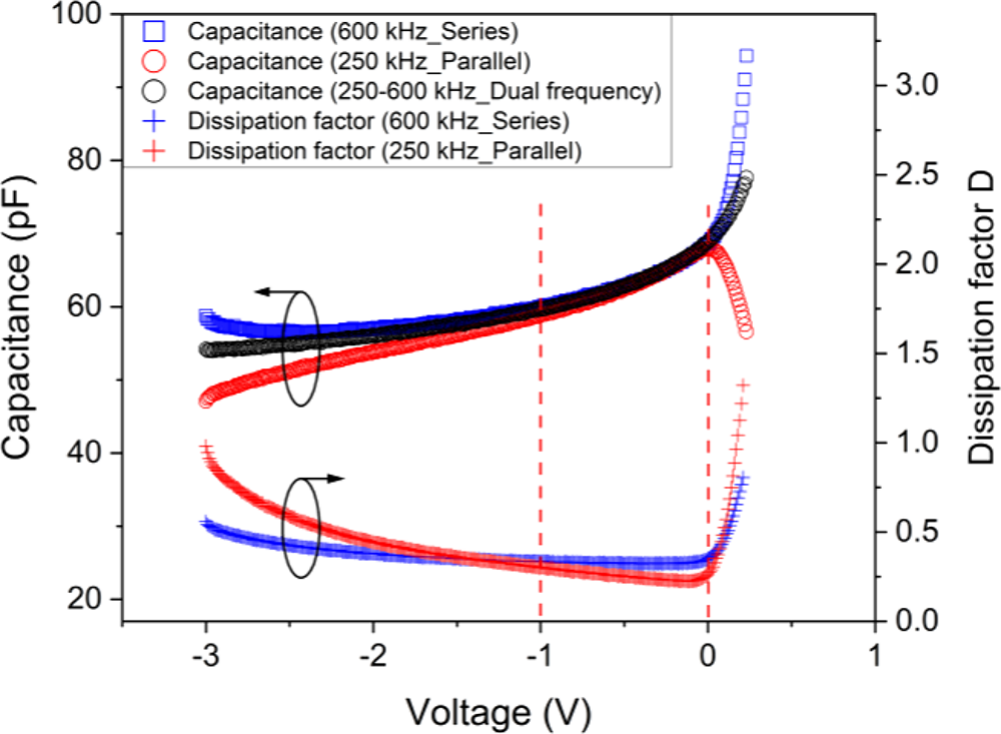
Left *y*-axis: *C*–*V* profile for selected single and dual frequencies. Right *y*-axis: Dissipation factor vs voltage for series (600 kHz) and parallel (250 kHz) configurations.

In Figure 4 we reported four capacitance sets of data obtained from series model at 600 kHz, parallel model at 250 kHz, and dual-frequency model at 250–600 kHz for the parallel model.

Once again, we can note that only in a limited voltage range, approximately between −1 and 0 V, are all capacitance data relatively coincident. Self-consistently, this is also the range where the dissipation factor is minimized, according to eq 3 and the discussion in ref (57) concerning the correlations between the *D*-factor and carrier type accuracy from the *C*–*V* profile.

The observed self-consistency confirms the correctness of the three circuit elements and of the series or parallel model in the appropriate frequency and bias ranges. Moreover, it guarantees the absence of any systematic error or spurious contribution to the measurement. Therefore, the observed nonlinearities of the 1/*C*^*2*^–*V* trend in the same measurement ranges (see Supporting Information) are not artifacts but are really due to nonuniform doping profile, as will be discussed in the following sections.

### 3.2 Doping Profile

The impurity concentration level is calculated by two distinct approaches. In the first one, it is directly evaluated from the experimentally obtained *C*–*V* plot (section 3.2.1), while in the second approach we used the Sentaurus-TCAD simulator for extracting the impurity concentration and gain additional insight into the experimental data (section 3.2.2).

#### 3.2.1 Doping Profile through Experimental *C*–*V*

From the measurement of the true capacitance vs applied voltage one can obtain a plot 1/*C*^2^–*V*, which provides some important parameters of the junction, such as built-in potential and doping density. When the doping profile is uniform, as typically happens for Schottky contacts on uniform bulk material, the plot is linear and the built-in diode potential corresponds to the intercept of 1/*C*^2^ on the voltage axis. In the case of a p–n junction, an effective doping density is again derived from the slope of the 1/*C*^2^–*V* plot,^57,62^ but one has to note that it is a combination of the profiles in the n- and p-sides of the junction. If the doping profiles are uniform and the depletion approximation can be applied, the relation between the net doping level in the n-type region, *N*_d_, and the net doping level in the p-type side of the junction, *N*_a_, is given by *N*_d_*x*_n_*= N*_a_*x*_p_, where *x*_n_(*x*_p_) is the depletion region width in the n-side (p-side) of the junction, and *x*_n_ + *x*_p_ = *x*_D_, where *x*_D_ is the total width of the depletion region. The capacitance of a reverse-biased junction, when considered as a parallel plate capacitor is given by^64^
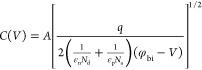
6awhere *ε*_n_(*ε*_p_) is the electrical permittivity of the n (p) layer (see Table 2), *q* is the electron charge, *φ*_bi_ is the built-in potential, and *A* is the junction area. Differentiating *C*–*V* or 1/*C*^2^–*V* with respect to voltage, gives
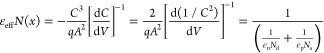
6bwhere ε_eff_ is an effective electrical permittivity and *N*(*x*) is the apparent doping profile of the p–n depletion region. If the junction is strongly asymmetric and *ε*_p_*N*_a_ ≫ *ε*_n_*N*_d_, *N*(*x*) and ε_eff_ represent the net doping profile and the electrical permittivity of the less doped side of the junction, respectively. In the present case, where the Ga_2_O_3_ side is less doped, it holds ε_eff_*N*(*x*) ≈ *ε*_n_*N*_d_ and
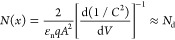
6cWhen the hypothesis of having a symmetric junction is not verified, the doping level of one side of the junction can be calculated by the 1/*C*^2^–*V* plot (see eq 6c) only if ε_n_ and ε_p_ and the doping level of the other side of the junction, assumed constant, are known.

**Table 2:** Main Parameters Used in the Simulation

Material	SnO	κ-Ga_2_O_3_
Doping (cm^–3^)	5.87 × 10^18^^39^	3.1 × 10^18^ (bulk, after thermal treatment)^39^ variable next to the junction
*E*_g_ (eV)	0.7^48^	4.9^31^
*m*_e_/*m*_0_ (*m*_h_/*m*_0_)	0.36^a^ (1.7^48^)	0.3^13^ (4.2^36^)
ε/ε_0_	18.8^48^	10.2^a^
μ_n_ (μ_p_) (cm^2^/V·s)	3.1^a^ (3.1^39^)	3^a^ (10^–6^ a)

a Assumed Values.

Similarly, the depletion region width, *x*_D_, can be calculated as
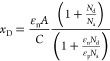
6dwhich reduces to
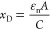
6eif  ≪ 1 and .

For the analyzed SnO/κ-Ga_2_O_3_ junction, in the SnO p-type layer both doping level^39^ and dielectric permittivity^48^ are higher than in the gallium oxide (n-side); therefore, the measured *N*(*x*) is expected to be mainly decided by the dopant concentration of κ-Ga_2_O_3_. Under the reasonable hypothesis of strongly asymmetric junction, we might assume that the doping density of the n-side region is provided by eq 6c and the width of the depletion region by eq 6e. In this case, the 1/*C*^2^*–V* plot is not linear; therefore, it is not possible to extract the built-in potential from a linear fit. To conclude, only an apparent *N*(*x*) density can be evaluated by eq 6c. The numerical derivative of the nonlinear 1/*C*^2^*–V* curve (eq 6a) resulted in scattered *N*_d_ vs *x*_D_ (net donors doping level vs depletion depth) values. Smoothing and analytical function fitting may reduce the fluctuations of the *C*–*V* plot and hence provide a smoother *N*_d_ vs *x*_D_, profile.

**Figure 5:**
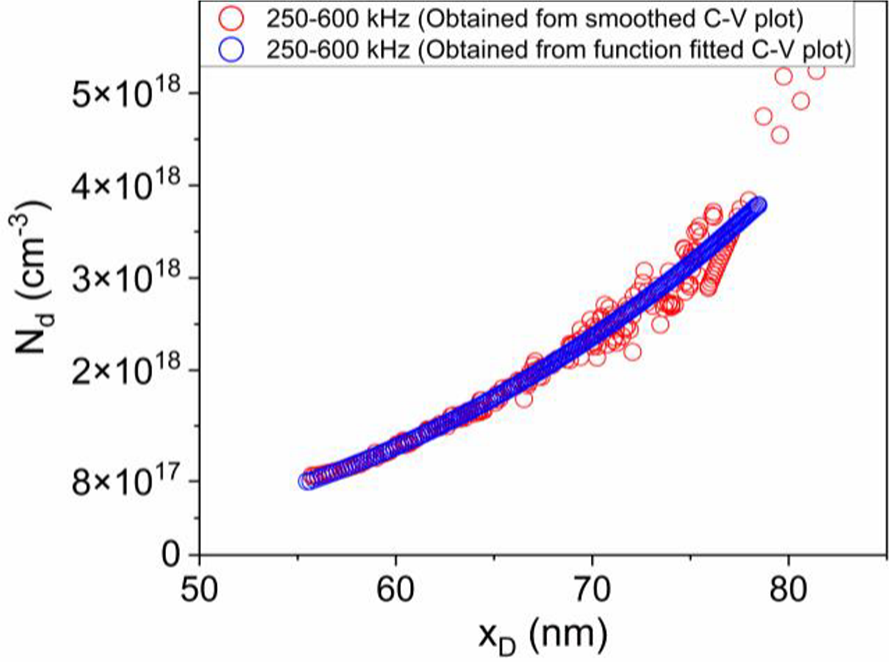
*N*_d_ vs *x*_D_ profile for dual frequencies using different approaches to smooth the *C*–*V* profile.

Figure 5 reports the net donor profile vs depletion depth obtained from eqs 6b and 6d using capacitance from the dual-frequency approach after data smoothing and function fitted *C*–*V* (in the voltage range of reliability of the experimental *C*–*V* data).

They resulted in excellent agreement with the fluctuating unsmoothed data (not shown here) and suggested a nonuniform impurity concentration across the depleted region, which has been completely attributed to the n-side of the junction. No evidence of nonuniformity in the SnO layers have been in fact observed in similar diodes.^48^ Moreover, in the following section 3.4, experimental data will be discussed that are consistent with the generation of doping nonuniformities in the κ-Ga_2_O_3_ layer during the deposition of the p-type SnO layer at 350 °C under oxygen plasma.

#### 3.2.2 Doping Profile Estimated by Numerical Modeling

**Figure 6:**
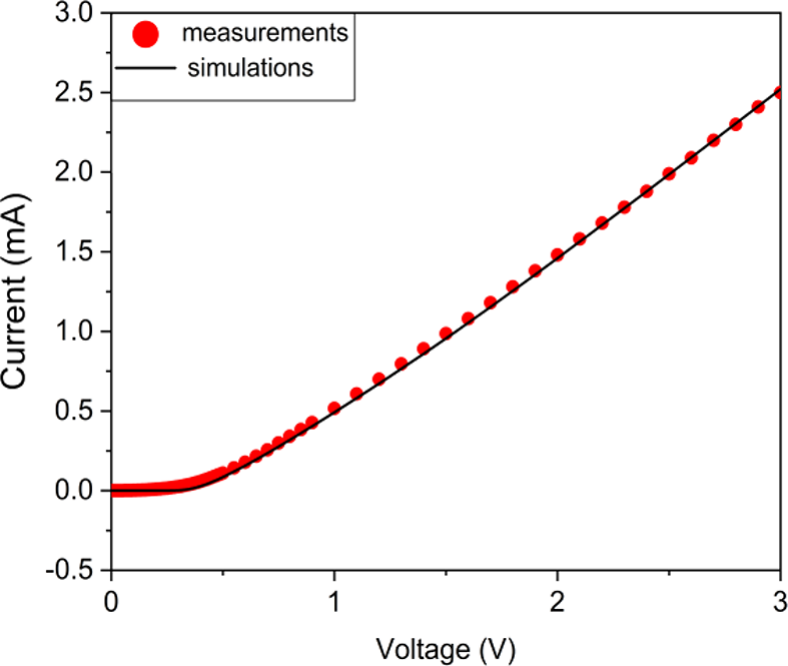
Measured and simulated *I*–*V* curves at RT.

To gain more insight about the dopant distribution in the n-type material, we implemented a three-dimensional model of the diode with the Synopsys Sentaurus-TCAD suite, as described in ref (65) for a p–i–n power diode and calculated the device response to the AC signal and the related frequency-dependent admittance. Since the software returns the parallel circuit response (see Figure 1C), the simulated capacitance was compared to the capacitance measured in the parallel configuration, *C*_p_, at the highest and lowest frequencies used, 250 and 600 kHz. The main parameters used for the simulations are listed in Table 2. Some properties were experimentally measured by Hall effect (electron mobility, *μ*_e(Ga_2_O_3_)_ and the doping concentration of κ-Ga_2_O_3_)^39^ or taken from previous works.^48^ A distributed specific series resistance of 0.24 Ω·cm^2^ at the base contact allowed us to obtain the match between the measured and simulated *I–V* (current–voltage) curve in the ohmic region, as shown in Figure 6.

The good match between the curves made us confident about the reliability of parameters used for the diode modeling. No other parameters were used to improve the match between the measurements and simulations.

It is worth noting that we also analyzed the effect of different mobility values in-plane and perpendicular to the junction. Such anisotropy in the mobility values originates from the formation of columnar domains in the κ-Ga_2_O_3_.^42^ It can be noted that the dominant transport mechanism for in-plane conduction is the variable range hopping, leading to low mobility values.^13^ In particular, considering that the mobility along a columnar domain can be at least 1 order of magnitude higher than the in-plane mobility^42^ but that the vertical transport across the SnO/κ-Ga_2_O_3_ interface may considerably be reduced by virtue of the interface defects, we assumed a mobility of 3 cm^2^/V·s for both orientations after having verified that neither the absolute mobility value nor its anisotropy significantly influenced the results of the simulation. The minor anisotropy role is probably related to the dominant horizontal current flow with respect to the vertical current flow for the planar geometry of the analyzed diode (Figure 1A).

**Figure 7:**
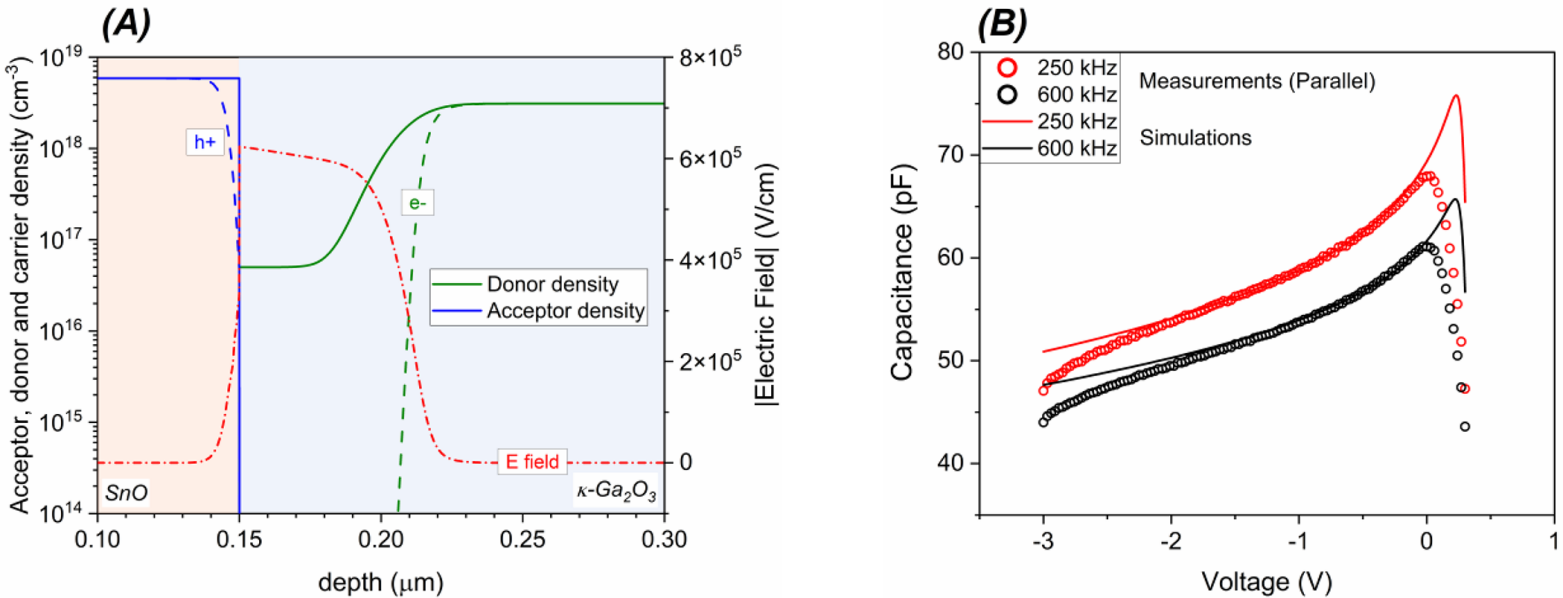
Acceptor, donor, and carrier profiles at equilibrium along a vertical line in the middle of the diode. Also shown is the absolute value of electric field in the junction region. The *x*-axis origin is taken at the surface of SnO. (B) Measured and simulated capacitance versus voltage curves at AC single frequencies of 250 and 600 kHz. The simulated capacitances are obtained with the diode doping profiles shown in (A).

The hypothesis that the nonlinear trend of the 1/*C*^2^–*V* curve is an effect of the heterointerface as in ref (66), taking a constant doping profile in the Ga_2_O_3_ side of the junction, resulted in a simulated capacitance not matching the experimental one. It was necessary to assume the doping profile shown in Figure 7A, where the actual dopant concentration on the n-side of the junction decreases following an error function trend from 3.1 × 10^18^ cm^–3^ in the κ-Ga_2_O_3_ bulk to 5 × 10^16^ cm^–3^ at the junction. In particular, as can be seen from the electric field profile in Figure 7A, even if the depletion region is mostly extending in the n-type material due to the asymmetrical doping of the p- and n-side, a nonzero electric field also extends over few nanometers in the SnO. As can be seen in Figure 7B, when a nonuniform error function distribution of dopant is selected, the simulated parallel capacitance is in very good agreement with the measured one, especially at the lowest frequency of the AC signal, which is the most meaningful set of data for the calculation of doping profile from the single-frequency parallel capacitance (see discussion in section 3.1.1). The choice of the doping profile is a most critical simulation factor, and only by postulating a very low doped interfacial layer on the n-side are the fitting results satisfying. Otherwise, the simulated *C*_p_ data deviated remarkably from the experimental ones.

**Figure 8:**
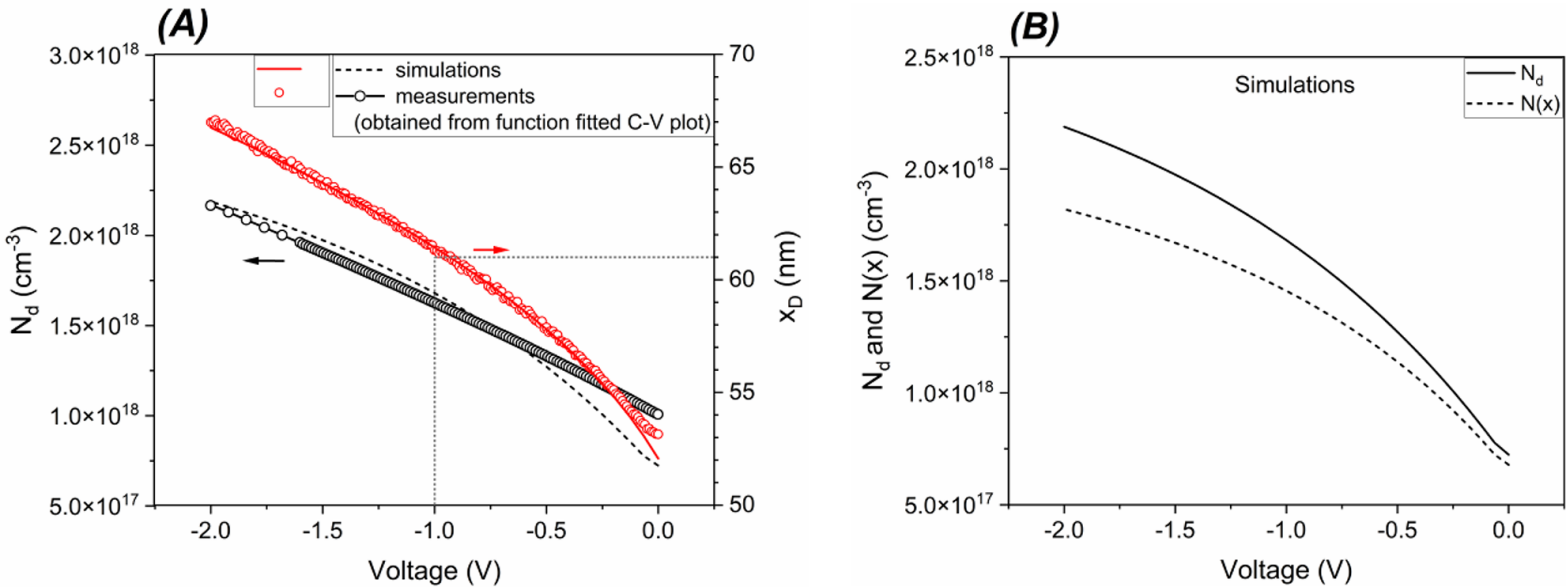
(A) Comparison between measured and simulated donor concentration profile, *N*_d_, and depletion region width, *x*_D_. Measured and simulated *N*_d_ have been calculated by eq 6b from the function fitted *C*_p_ at 250 kHz and from the corresponding simulated capacitance, respectively (both capacitance profiles are shown in Figure 7B). The obtained *N*_d_ values are then inserted in eq 6d to calculate the corresponding *x*_D_. (B) Simulated doping profiles *N*_d_ and *N*(*x*) versus the bias voltage calculated by eqs 6b and 6c, respectively (see text for additional details).

In spite of the observed spatial nonuniformity of the net doping density, the largest fraction of the depleted region is confirmed to fall in the n-type side of the heterojunction. As explained in section 3.1.1, at low frequency the parallel *C*_p_ is expected to approach well the real capacitance, *C*, i.e., the depletion capacitance. Then, as a self-consistency test, the *N*_d_ doping density of the κ-Ga_2_O_3_ layer and the *x*_D_ depletion width are calculated by eqs 6b and 6d either from the simulated *C*_p_ data or from the function fitting of the experimental capacitance values (see Section 3.2.1), both evaluated at 250 kHz. In particular, for both sets of data, the *N*_d_ density was calculated at each applied potential value (*V*_a_) from eq 6b and then inserted in eq 6d together with the corresponding capacitance value *C*(*V*_a_) to obtain *x*_D_. Their dependences on the applied voltage are compared in Figure 8A: the simulated *N*_d_ data well reproduce the experimental ones for voltages below −0.5 V and deviate for bias voltages approaching 0 V due to the slightly worse match between simulated and experimental *C*_p_ in the range −0.5 to 0 V (see Figure 7B). In Figure 8B, instead, the voltage dependence of *N*_d_ obtained from eq 6b is compared to the behavior of *N*(*x*) calculated by eq 6c.

It is apparent that the more negative the reverse bias, the higher the modulated charge, but at the same time, the hypothesis *ε*_p_*N*_a_ ≫ *ε*_n_*N*_d_ is no longer completely fulfilled, causing the two profiles to diverge (see Figure 8B). The relative error reaches about 16% at −2 V. Calculating *N*_d_ with eq 6b is thus to be preferred for the studied diode, as we will discuss in the following.

### 3.3 Electron and Holes Responses to the Applied Bias

**Figure 9:**
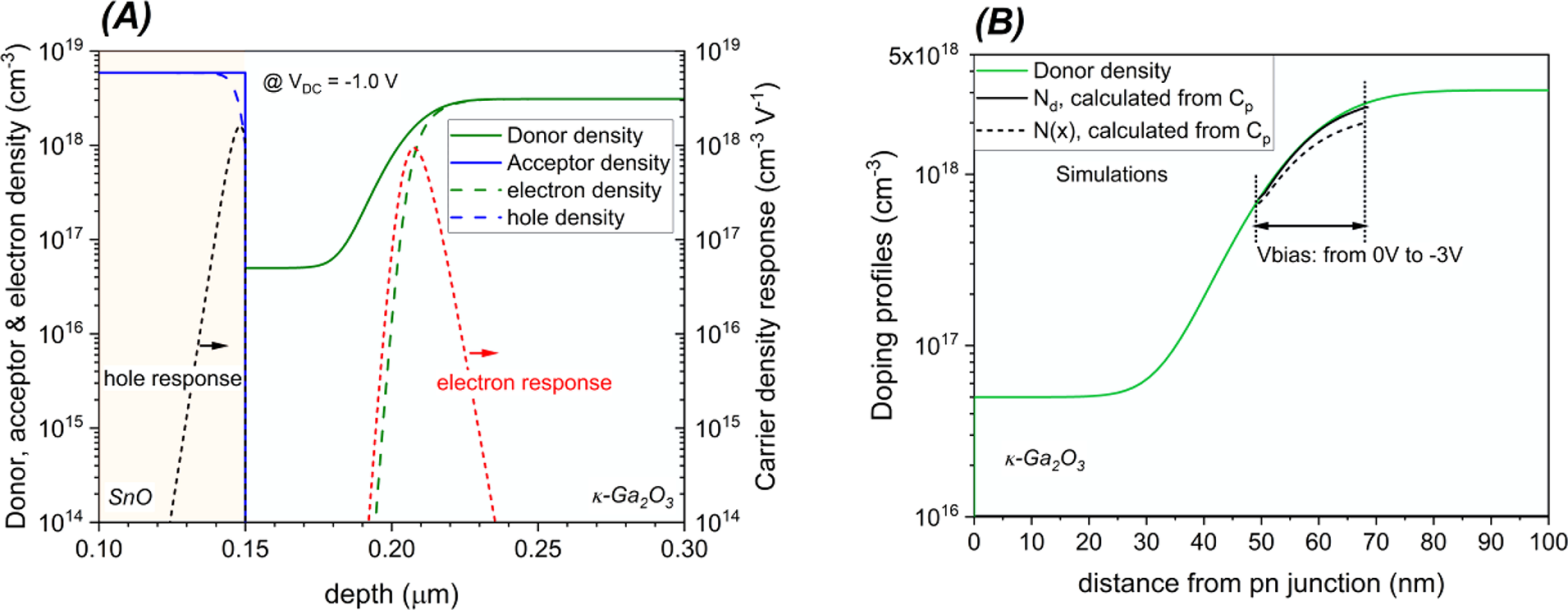
(A) Left *y*-axis: acceptor, donor, carrier profiles at a reverse voltage bias of −1 V. Right *y*-axis: hole and electron responses to the AC signal (frequency of 250 kHz). The *x*-axis origin is at the surface of SnO. (B) Actual donor density profile in the simulated diode, apparent impurity concentration profile, *N*(*x*), and n-side doping profile, *N*_d_, versus depth calculated from the simulated parallel capacitance *C*_p_ (AC signal frequency of 250 kHz).

Another significant piece of information that can be extracted through the simulation is the electron and hole response to the applied bias (Figure 9A).

As shown in Figure 9A, the electrons and holes are responding well to the applied bias in the zones in proximity of the depleted region edges: as an example, the peak response is at about 58 nm (electrons) and 2 nm (holes) from the p–n junction for a reverse bias of −1 V. As can be seen in Figure 8A, the sum of these values is quite close to the depletion region depth *x*_D_ ≈ 61 nm calculated at −1 V from both the simulated and measured capacitance at 250 kHz.

To test the accuracy of the procedure used to calculate the doping profile from the simulated *C*_p_, we compared the calculated *N*_d_ from eq 6b with the actual donor concentration defined in the simulation (green solid curve in Figure 9B). To this purpose, the depletion region has been calculated with eq 6e.

As shown in Figure 9, *N*_d_ plotted as a function of the n-side depletion region width, *x*_n_, overlaps very well with the actual doping profile provided by the simulation, if *x*_n_ ≈ *x*_D_ – 2.5 nm is considered. In other words, the extracted doping profile reproduces well that of the region where electrons are responding to the AC signal (see Figure 9A). *N*(*x*) vs *x*_n_, instead, underestimates the n-side doping, as it also includes the contribution of the p-side of the junction, which is no longer negligible. The 2.5 nm value that ensures the optimal alignment between *N*_d_ and the actual doping profile defined in the simulation is consistent with the extension of the depletion region in the p-side of the junction, as reported in Figure 9A.

**Figure 10:**
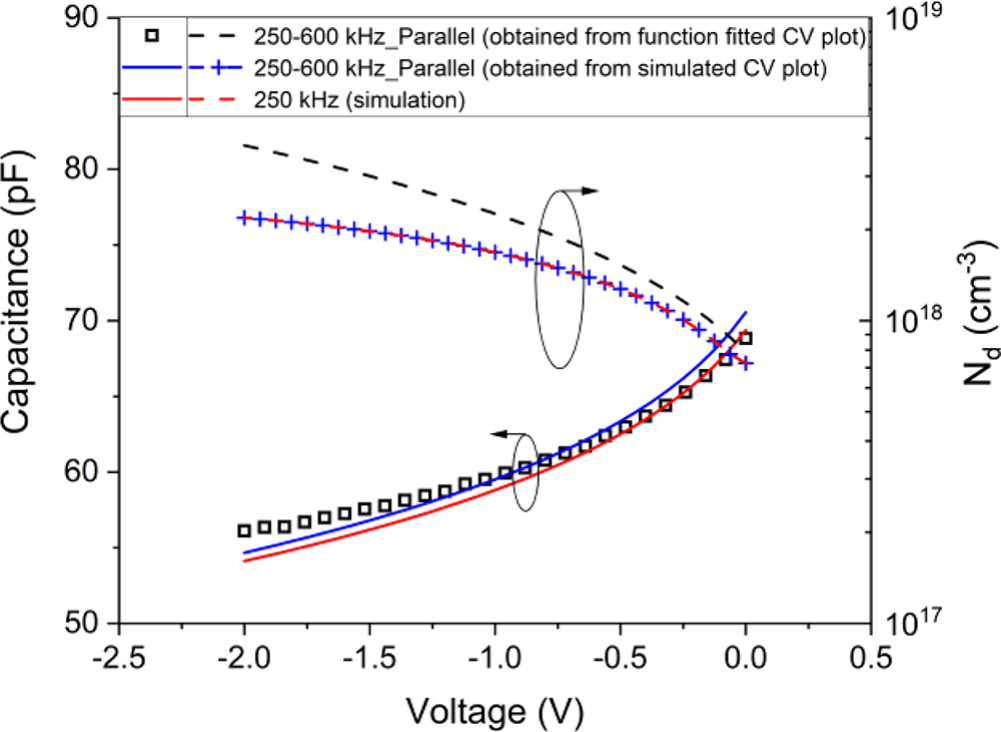
Left *y*-axis: measured and simulated dual-frequency capacitances and simulated capacitance at 250 kHz versus the bias voltage. Right *y*-axis: corresponding n-side doping profile, *N*_d_, caculated by eq 6b (see text).

To complete the analysis, the parallel dual-frequency capacitance at 250–600 kHz is calculated by eq 5 from the simulated *C*–*V* curves and compared with the function-fitted experimental dual-frequency capacitance and the simulated capacitance at 250 kHz (Figure 10).

The *N*_d_ values taken from simulated single- and dual-frequency *C*–*V* curves perfectly overlap; therefore, for the studied device, both the parallel single- and dual-frequency approach appear to be accurate enough to derive the doping from the *C*–*V* curve through the simulation.

On the other hand, the *N*_d_ values from the experimental and simulated dual-frequency *C*–*V* curves tend to diverge, particularly for increasing negative bias (Figure 10). This might be related to summed single-frequency uncertainties in the dual-frequency approach. Nevertheless, *N*_d_ calculated from *C*_p_ at 250 kHz, can be considered a reliable indication of the real dopant distribution in the κ-Ga_2_O_3__._ Major sources of uncertainty come from the fluctuations of the experimental *C*–*V* which affects the calculation of experimental dual-frequency capacitance from single-frequency curves.

The approach presented in this work was applied to our κ-Ga_2_O_3_ based diodes, but it is conceptually extendable to other p–n junctions. It is a general and flexible approach that may turn out useful for analysis of different junction types, whether the relative contributions of the shunt or series resistance.

### 3.4 Electrical Properties κ-Ga_2_O_3_ after Overgrowth of SnO

In a planar structure, like the SnO/κ-Ga_2_O_3_ heterojunction of this work, the series resistance between the two electrodes (see Figure 1) strongly influences the in-plane transport, as shown in ref (39). The multifrequency *C*–*V* approach here proposed, coupled to accurate device simulation, enables a complete and reliable assessment of these heterojunctions. It also allows us to estimate how the properties of the κ-Ga_2_O_3_ starting layer are altered by the SnO overgrowth, that is, interface and bulk properties after the PAMBE process.

Both the experimental *C*–*V* measurements and simulations point toward a nonuniformity of the doping profile within the depletion region of the n-type material (κ-Ga_2_O_3_). To understand the origin of the change of κ-Ga_2_O_3_ functional properties during the deposition of the p-type SnO layer on the n-type layer, a treatment that simulates the MBE deposition of SnO has been performed (GSH cycle) on Si-doped κ-Ga_2_O_3_ layers. The GSH cycle reproduces on samples 2, 3, and 4 of Table 1 the procedure applied for fabrication of the diode via SnO deposition (as for sample 1), and consists of the four steps i-iv described in the Experimentl section.

**Figure 11:**
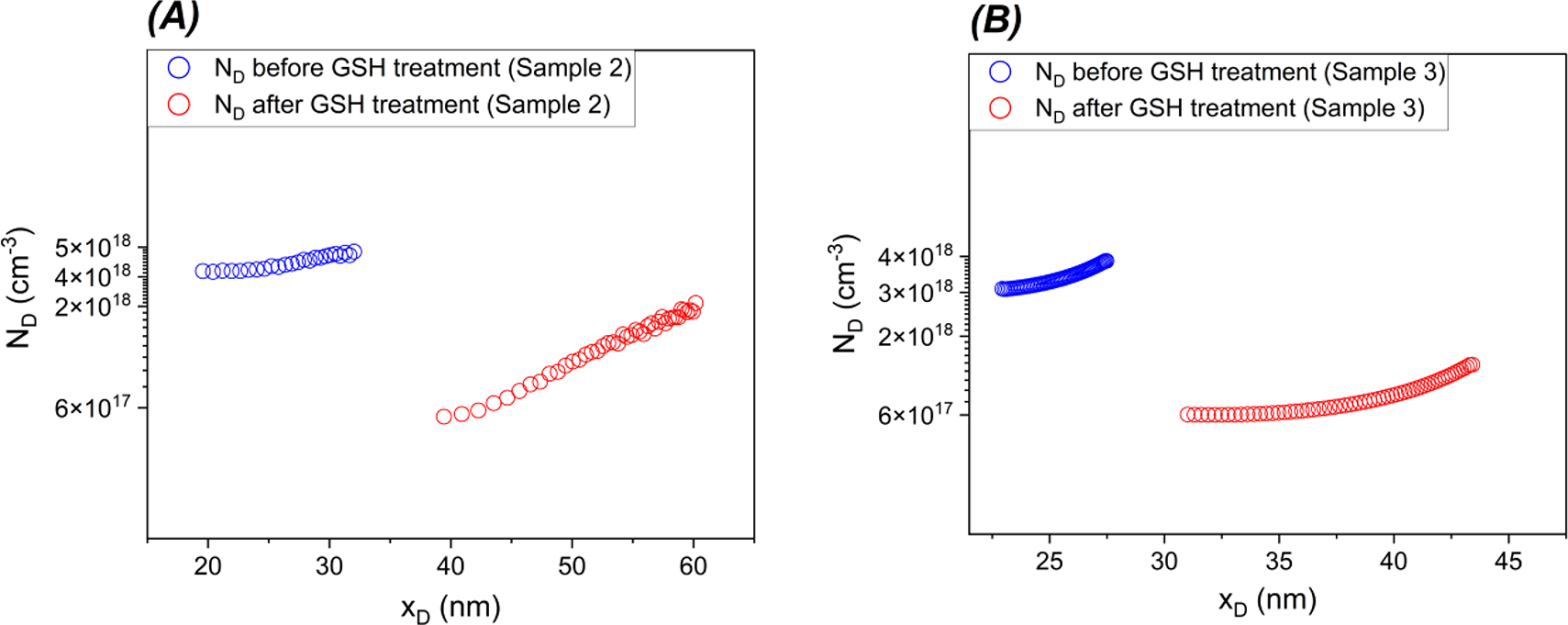
*N*_d_ vs *x*_D_ profiles before and after GSH treatment. (A) Sample 2: Hg-probe measurement used to calculate the doping density. (B) Sample 3: Doping density obtained from the function fitted *C*–*V* curve of the Schottky diode. The different depleted depth is a consequence of the different net donor concentration of the material before and after thermal treatment. In all cases, the measurements were performed in reverse bias, in a range where the dissipation factor was low.

The properties of pristine samples were compared with those of samples submitted to the GSH cycle. We performed Hg-probe *C*–*V* measurement at RT on sample 2 (method described in ref (67); results reported in Figure 11A) or measured the *C*–*V* of the Schottky diode fabricated on sample 3, using eqs 6c and 6e to extract the doping profile (Figure 11B). Hg-probe *C*–*V* data of sample 3 are reported in the Supporting Information. The change of the doping profile near the surface (see Figure 11A,B) clearly indicates that a significant concentration drop occurs near the surface as a consequence of the thermal treatment.

This postanneal behavior resembles that of the heterojunction shown in Figure 5, and the predictions of the modeling (Figure 7, Figure 10), which suggests that it is the thermal treatment, partly in oxygen plasma, that causes the doping reduction in proximity of the surface, even without performing the SnO deposition. Such GSH-related reduction of the doping density near the surface may be beneficial in modulation of the drift region in the vertical devices in which the low donor density is preferable.^14^

**Figure 12:**
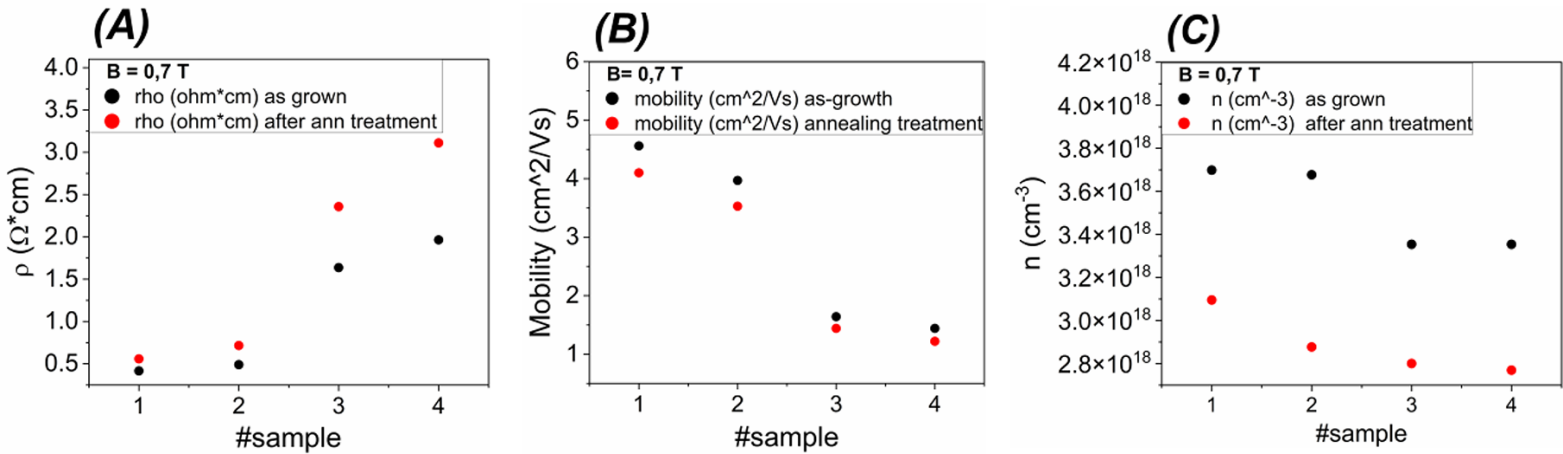
Resistivity (A), Hall mobility (B) and Hall density (C) measured in the van der Pauw configuration at RT on a set of as-grown samples (black symbols) and for each of them a second piece of the same epitaxial deposition (red symbols) which was submitted to GSH thermal treatment (see text). The label of each sample is reported on the abscissa axis (thickness of the samples around 500 nm). Error limit in the resistivity and Hall data are approximately equal to 5–10% and 10%, respectively.

We completed the investigation by measuring the RT resistivity and Hall effect on four s-grown samples exposed to a GSH cycle. The results of this in-plane transport characterization are reported in Figure 12.

An increment of resistivity and a decrease of both Hall mobility and average free carrier density was detected at RT in the GSH treated samples with respect to the pristine ones.

The variation of the electrical properties of κ-polymorph after heating above RT in a vacuum have first been reported in ref (13), where a possible role of hydrogen out-diffusion or a nonequilibrium occupancy of deep levels was put forward. The effects of the GSH treatment corroborate this view. It is very likely that also in the SnO/κ-Ga_2_O_3_ diode here investigated the observed doping reduction on the n-side of the junction results from the heating of the sample prior and during the deposition of SnO.

This subsurface doping reduction is consistent with a diffusion process and is compatible either with donor species escape from the layer or with in-diffusion or activation of a certain concentration of acceptors. Among the possible phenomena leading to the observed alteration of the doping profile, the thermally activated out-diffusion of hydrogen is certainly a plausible hypothesis; in fact, interstitial hydrogen is expected to be a shallow donor in κ-Ga_2_O_3_, as already predicted for β-Ga_2_O_3_. Hydrogen can derive from the precursors and the carrier gas in κ-Ga_2_O_3_ layers.^13,39^ In κ-Ga_2_O_3_ layers grown using H_2_ carrier gas but without providing an additional silane (SiH_4_) flow, resistivity of about 10^5^ Ω·cm was typically obtained.^13,42^ This implies that only a minor fraction of molecular hydrogen gas acts as a shallow donor, but we cannot exclude that a much more effective hydrogen incorporation may take place when the silane gas cracks at the surface of the growing κ-Ga_2_O_3_ layers, with release of atomic H. Therefore, it is plausible that both atomic hydrogen and silicon can contribute to establish the n-type conductivity.

Furthermore, it is known that hydrogen in beta gallium oxide may play a double role: give rise to shallow donors and decorate (passivate) Ga vacancies. Should a similar behavior be true also for the κ-polymorph, the hydrogen out-diffusion may produce a reduction of shallow donors as well as a reactivation of deep acceptor energy levels (e.g., ref (68)).

Also, the silicon passivation due to the possible formation of a (V_Ga_–Si) complex defect may be considered as another factor relevant to the drop of donors in the n-side of the junction. This is possible if a Si donor shifts in interstitial position between two Ga vacancies, as hypothized for the beta-polymorph.^69^ The formation of such a defect complex could be triggered by hydrogen out-diffusion. The reduction of oxygen vacancies, which are deep donors in κ-Ga_2_O_3_, induced by the exposure to oxygen plasma in the first stage of PAMBE growth of SnO^39^ could also play some role.

Lastly, it cannot be excluded that the nonequilibrium occupancy of deep levels could affect the change of transport properties shown in Figure 12, as suggested in ref (13). In fact, in sample 2, the 40% increase in resistivity and the 15% decrease of mobility of the heated vs as-grown sample cannot be simply justified by the doping profile from surface down to the bulk and points at an increased compensation.

## 4 Conclusion

A comprehensive characterization of planar diodes fabricated by depositing p-type SnO on n-type κ-Ga_2_O_3_ was presented. Because of this device layout, the analysis of capacitance–voltage measurements may follow different models: series or parallel configuration, depending on the measurement frequency. Analysis of the *C*–*V* profiles measured at four different frequencies showed that the highest frequency, for series configuration, and the lowest frequency, for the parallel model, agree well with the dual-frequency method. The dual-frequency method, however, can provide the most reliable *C*–*V* profiles, using pairs of well separated frequencies (e.g., 250–600 kHz) and thus give an accurate description of the characteristics of the diodes. Remarkably, in the bias range in which the dissipation factor is minimized, all *C*–*V* profiles from the series, parallel, and dual-frequency methods are practically coincident, supporting the proposed three-element equivalent circuit and the absence of systematic errors in the measurement. For the practical SnO/κ-Ga_2_O_3_ case studied in this work, it was found that the impurity concentration aside from the p/n interface is not homogeneous, especially in the n-type material. Because the junctions were not strongly asymmetric, only an apparent doping profile can be obtained from the experimental 1/*C*^2^–*V* plots. Therefore, we used Sentaurus simulation in order to estimate the impurity concentration within the individual n- and p-type layers. The significant net carrier concentration drops within the n-type κ-Ga_2_O_3_ layer, close to the p/n interface, was primarily ascribed to hydrogen out-diffusion, with a possible simultaneous passivation of silicon, induced by the heating of the epilayer prior and during SnO deposition. To support this hypothesis, the same annealing conditions were applied to simple n-type κ-Ga_2_O_3_ epilayers, and the subsequent electrical measurements confirmed a substantial increase of the film resistivity, compatible with a reduction of the doping near the surface. Other possible effects, such as saturation of O-vacancies during PAMBE growth, are believed to play a minor role.

This study also demonstrated the instability of the electrical properties of κ-Ga_2_O_3_ upon heating in certain environmental conditions, which must be taken into consideration when designing the fabrication steps of κ-Ga_2_O_3_-based diodes.

## Supporting Information Available

Additional information about result of Raman spectroscopy measurement of the SnO layer, capacitance vs frequency (*C*–*f*) plot, 1/*C*^2^–*V* plot for series and parallel configurations, *C*–*V* profile for the dual frequency in series configuration, *N*_d_–*x*_D_ profile obtained from series configuration, additional Hg-probe measurements (PDF)
