# Supplementary material for: Interfacial Properties of the SnO/κ-Ga2O3 p-n Heterojunction: A Case of Subsurface Doping Density Reduction via Thermal Treatment in κ-Ga2O3
Source: ACS Appl Mater Interfaces. 2023 Sep 21;15(39):45997–6009. doi: 10.1021/acsami.3c08841 (PMC10561148; doi:10.1021/acsami.3c08841)
Supplement: Supplementary file 1 — am3c08841_si_001.pdf [file am3c08841_si_001.pdf]

Supporting Information

Interfacial Properties of the SnO/κ-Ga_2O_3 p-n Heterojunction: A Case of Subsurface Doping Density Reduction via Thermal Treatment in κ-Ga_2O_3

Payam Rajabi Kalvani[1], Antonella Parisini*[1], Giovanna Sozzi[2], Carmine Borelli[1], Piero Mazzolini[1,3], Oliver Bierwagen[4], Salvatore Vantaggio[1], Kingsley Egbo[4], Matteo Bosi[3], Luca Seravalli[3], Roberto Fornari*[1,3]*

1. University of Parma, Department of Mathematical, Physical and Computer Sciences, Parco Area delle Scienze 7/A, 43124, Parma, Italy
2. University of Parma, Department of Engineering and Architecture, Parco Area delle Scienze 181A, 43124 Parma, Italy
3. IMEM-CNR, Institute of Materials for Electronics and Magnetism, Parco Area delle Scienze 37/A, 43124, Parma, Italy
4. Paul-Drude-Institut für Festkörperelektronik, Leibniz-Institut im Forschungsverbund Berlin e.V., Hausvogteiplatz 5-7, 10117 Berlin, Germany

*Corresponding Authors: payam.rajabikalvani@unipr.it; antonella.parisini@unipr.it; roberto.fornari1@unipr.it;

1. Raman spectra of p-side of the junction (*SnO*)

To further confirm the growth of *SnO* on κ-Ga_2O_3 and to clarify the phase composition beyond the XRD scan, the result of room temperature Raman spectroscopy measurement of the *SnO* layer is reported in Figure S1. The measured Raman spectra of the *SnO* layer shown in Figure S1 mainly consists of B_{1g} (~113 cm^{-1}) and A_{1g} (~211 cm^{-1}) peaks due to *SnO* and weak *O*-rich secondary peaks of Sn_3O_4 coexisting with the *SnO* phase as previously observed [3] due to slightly *O*-rich growth. These *O*-rich Sn_3O_4 phases do not influence the diode properties of SnO/Ga_2O_3 based diodes [4].

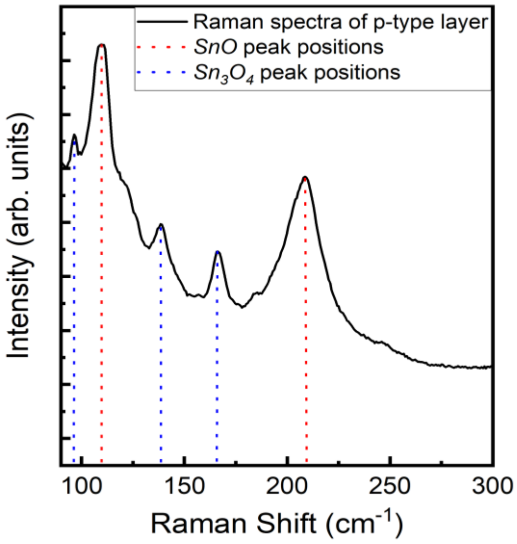

Figure S1. Raman analysis taken from the p-side of the junction at room temperature. Red and blue dots are corresponding to peak positions of *SnO* and Sn_3O_4 respectively.

2. Capacitance vs. frequency (*C-f*) in series and parallel configurations

Figure S2, reports data of capacitance vs. frequency (*C-f*) measurement for both series and parallel configurations, measured for the SnO/κ-Ga_2O_3 diode, at room temperature.

Note that increasing the frequency in series configuration (C_s-f measurement), correlated to decreasing the frequency in the parallel configuration (C_p-f measurement). The capacitance value for series configuration (C_s) at the highest frequency (1 MHz) is equal to the capacitance value for parallel configuration (C_p) at the lowest frequency (100 Hz).

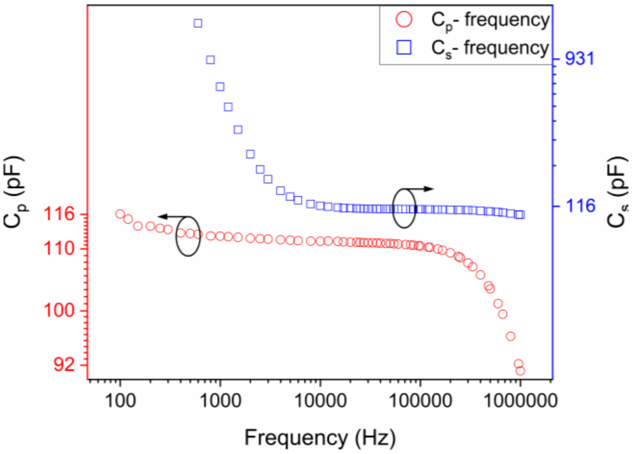

Figure S2. *Left y-axis:* C_p vs. frequency, *Right y-axis:* C_S vs. frequency profiles at room temperature. Symbols: blue squares are for series configuration; red circles are for parallel configuration.

3. Curvature in the *1/C*²*-V* plot

Such a curvature in the $1/C^2$-V plots (Figure S3), derives from the non-constant doping profile in the n-side of the junction.

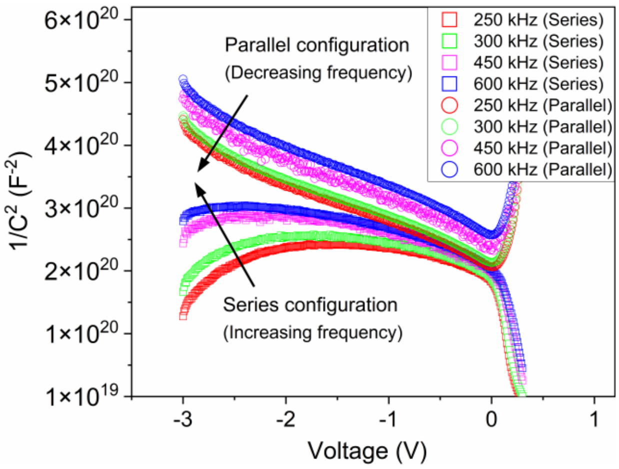

Figure S3. $1/C^2$ *vs.* V profile at four different frequencies. Symbols: squares are for series configuration; circles are for parallel configuration. Red: 250 kHz; green: 300 kHz; pink: 450 kHz; blue: 600 kHz.

4. Dual-frequencies in the series configuration

The dual-frequency method is used to measure the true capacitance [1,2]. Such method can be applied to both series and parallel configurations. In order to verify the equivalence of the two alternative procedures, and exclude systematics errors in the measurement, as a reproducibility test, we

calculated the true capacitance from the dual frequency in the series configuration through equation (*S1*):

$$C = \frac{\omega_2{}^2 C_{s2} - \omega_1{}^2 C_{s1}}{\omega_2{}^2 - \omega_1{}^2} \qquad (S1)$$

where C_{s1}, C_{s2} are capacitance values measured at the first and second frequencies (ω_1 and ω_2), for the series model respectively.

The dual-frequency method in the series configuration was applied for the following pairs of frequencies: 250-300 kHz, 250-450 kHz, 250-600 kHz, 300-450 kHz, 300-600 kHz, 450-600 kHz (Figure S4), and the results are relatively similar to the true capacitance value and obtained from the dual-frequency in the parallel configuration (Figure 3. of the manuscript).

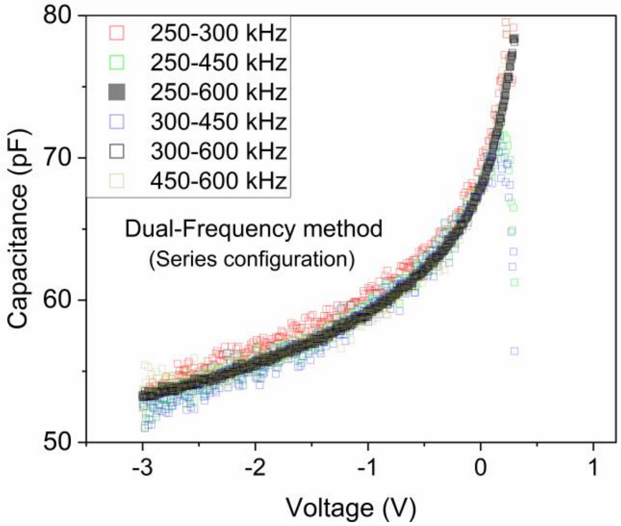

Figure S4. *C-V* profile of the dual-frequency method for series configuration; note the good matching of measurements at 250-600 kHz (Black filled squares symbols).

Negligible differences appear, as expected, indicating the uncertainty in the *C-V* experimental curves. Similar to the *C-V* profile obtained from the dual-frequency in the parallel configuration, the frequency pair 250-600 kHz, provide "optimal" *C-V* profiles with the highest signal-to-noise ratio.

Thus, regardless of the series or parallel configuration which we consider for calculation of the true capacitance, the pair of frequencies with the largest difference, provide the most accurate values (lowest fluctuations) and both configurations are giving the same results for the three elements model.

5. Doping profile obtained through the dual-frequency in series configuration

The doping density extracted from the true capacitance in series configuration for sample (1) is shown in Figure S5, which has similarities with the doping profile obtained from the derivative of the true capacitance in the parallel configuration (See figure 5 of the article). This can be another proof of matching between series and parallel configurations for calculation of the real capacitance and relevant doping profile by the dual frequency approach.

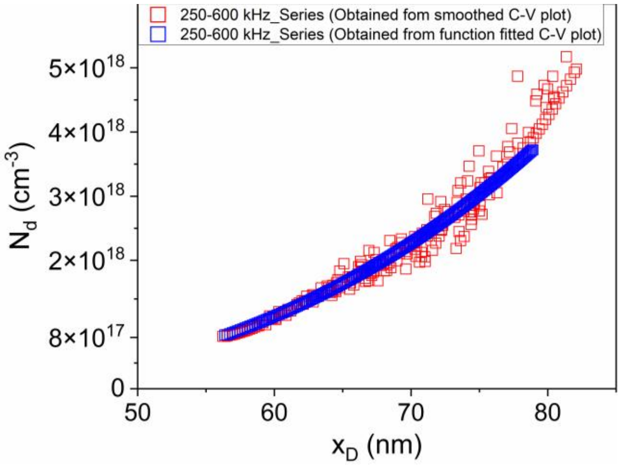

Figure S5. *N_D vs. x_D* profile obtained from the derivative of the true capacitance in the series configuration.

6. Further evidence of subsurface donor density reduction in *κ-Ga_2O_3*

6.1. Hg-probe measurement of sample 3

As a further proof of the subsurface donor density reduction in *κ-Ga_2O_3*, the doping density of sample 3 was also evaluated by the mercury probe measurement (Figure S6).

The observed trend in the doping density measured by Hg-probe, also demonstrates subsurface donor density reduction in *κ-Ga_2O_3* after Growth Simulation Heating (GSH) treatment.

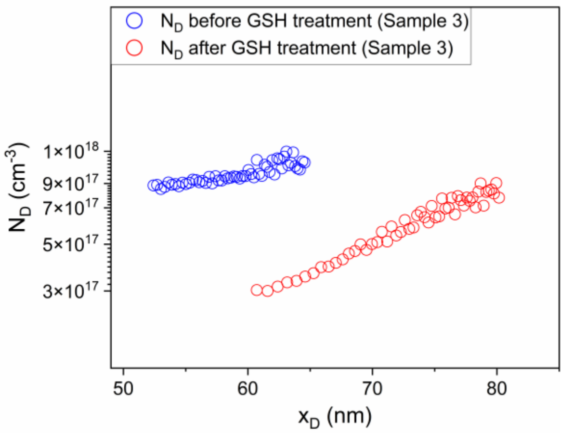

Figure S6. N_d *vs.* x_D profiles before and after GSH treatment measured by *Hg*-probe analysis for sample 3.

6.2. Schottky diodes with different sizes

In addition to the Schottky diode presented in section 3.4 of the article (Schottky diode made on sample 3), other Schottky diodes with similar (200µm×200 µm) and different (150µm×150 µm) dimensions were also studied in order to evaluate the subsurface doping profile (Figure S7).

In Figure S7, the net donors density profile in samples of different size *(A)* and *(B)* follow trend similar to those of the samples presented in the article, which further confirms the occurrence of subsurface donor reduction in the *κ-*Ga_2O_3 after thermal treatment.

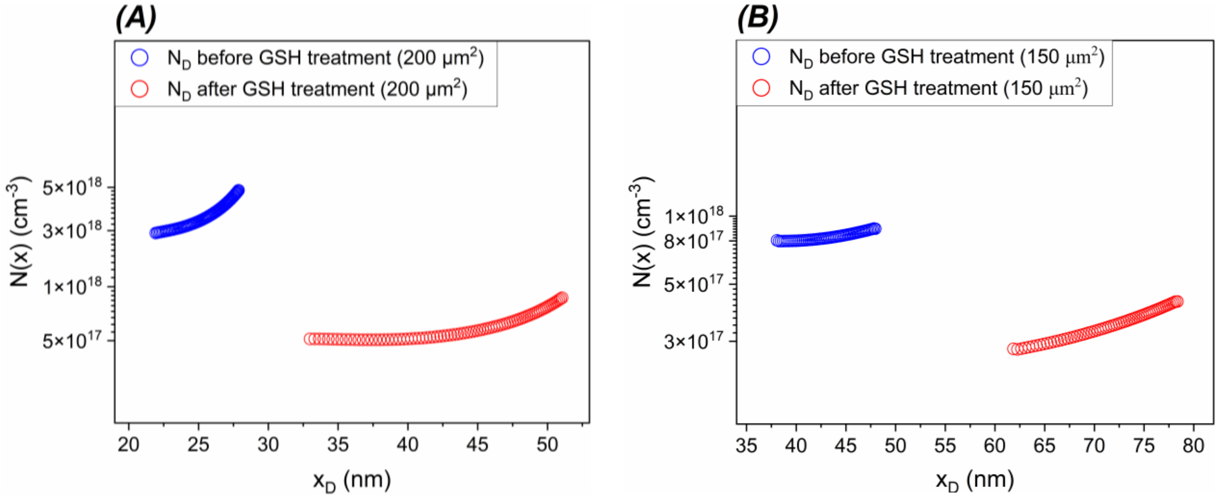

Figure S7. N_d *vs.* x_D profiles obtained from the function fitted *C-V* curve of the Schottky diode before and after GSH treatment in square shape samples with dimensions: ***(A).*** 200×200 µm^2, and ***(B)***. 150×150 µm^2. Symbols: blue circles are for the as grown samples (before GSH treatment), and red circles are for samples after the GSH treatment.

References:

(1) Lo/nnum, L. F.; Johannessen, J. S. Dual-Frequency Modified C/V Technique. *Electron. Lett.* **1986**, *22* (9), 456. https://doi.org/10.1049/el:19860310.

(2) Yang, K. J.; Chenming Hu. MOS Capacitance Measurements for High-Leakage Thin Dielectrics. *IEEE Trans. Electron Devices* **1999**, *46* (7), 1500–1501. https://doi.org/10.1109/16.772500.

(3) Egbo, K.; Luna, E.; Lähnemann, J.; Hoffmann, G.; Trampert, A.; Grümbel, J.; Kluth, E.; Feneberg, M.; Goldhahn, R.; Bierwagen, O. Epitaxial Synthesis of Unintentionally Doped *p* - Type SnO (001) via *Suboxide* Molecular Beam Epitaxy. *J. Appl. Phys.* **2023**, *133* (4), 045701. https://doi.org/10.1063/5.0131138.

(4) Tetzner, K.; Egbo, K.; Klupsch, M.; Unger, R.-S.; Popp, A.; Chou, T.-S.; Anooz, S. B.; Galazka, Z.; Trampert, A.; Bierwagen, O.; Würfl, J. SnO/β-Ga_2O_3 Heterojunction Field-Effect Transistors and Vertical p–n Diodes. *Appl. Phys. Lett.* **2022**, *120* (11), 112110. https://doi.org/10.1063/5.0083032.
